# Exosome mediated Tom40 delivery protects against hydrogen peroxide-induced oxidative stress by regulating mitochondrial function

**DOI:** 10.1371/journal.pone.0272511

**Published:** 2022-08-11

**Authors:** Nasif Sayeed, Kiminobu Sugaya

**Affiliations:** Burnett School of Biomedical Sciences, College of Medicine, University of Central Florida, Orlando, Florida, United States of America; University of Nebraska-Lincoln, UNITED STATES

## Abstract

Mitochondrial dysfunction is a hallmark of neurodegeneration. The expression level of Tom40, a crucial mitochondrial membrane protein, is significantly reduced in neurodegenerative disease subjects. Tom40 overexpression studies have shown to protect the neurons against oxidative stress by improving mitochondrial function. Thus, successful delivery of Tom40 protein to the brain could lead to a novel therapy for neurodegenerative diseases. However, delivering protein to the cell may be difficult. Especially the blood-brain barrier (BBB) is a big hurdle to clear in order to deliver the protein to the brain. In the current study, we engineered exosomes, which are the extracellular vesicles of endosomal origin, and able to cross BBB as delivery vehicles packing human Tom40. We found Tom40 protein delivery by the exosome successfully protected the cells against hydrogen peroxide-induced oxidative stress. This result suggests that exosome-mediated delivery of Tom40 may potentially be useful in restoring mitochondrial functions and alleviating oxidative stress in neurodegenerative diseases, such as Alzheimer’s and Parkinson’s diseases.

## Introduction

Progressive neuronal damage with a debilitating cellular response is a principal characteristic of common neurodegenerative diseases. Alzheimer’s disease (AD) is the most common neurodegenerative disease affecting more than 6 million Americans at present [1]. It is linked with cognitive impairment leading to dementia. Parkinson’s disease (PD) is the second most common neurodegenerative disease characterized by movement disorder and non-motor symptoms [2]. Defective glucose utilization and disrupted energy metabolism are frequent in neurodegenerative diseases [3]. Neurons vulnerable to degeneration demand more energy for structural and functional sustainability. Dysfunctional mitochondria, the oxidative energy generators of a cell, can therefore exert metabolic stress on neurons resulting in further degradation [4]. Several pieces of evidence depict that mitochondrial dysfunction either precedes the disease pathology or exacerbates the pathogenesis [5, 6]. The deficiency of major enzymes involved in glycolysis and tricarboxylic acid cycle (TCA) correlates well with the disease progression [7]. Reduction in brain glucose metabolism predicts the possible progression of mild cognitive impairment (MCI) in patients to a definite AD [8]. These defects in mitochondria of AD and PD brains cause structural damage to the organelle resulting in compromised bioenergetics, calcium dyshomeostasis, mtDNA integrity loss, and synaptic dysfunction [6]. Hence, mitochondrial dysfunction is a crucial factor in neurodegenerative diseases and is chiefly responsible for generating oxidative stress in neurons.

Oxidative stress, a prominent feature in neurodegenerative diseases, occurs when cellular capacity fails to scavenge overproduced oxygen-containing free radicals or reaction products of such moieties that are often called reactive oxygen species (ROS). Defective mitochondria, during the production of ATP, often become the potential source of ROS which in turn contributes more to mitochondrial dysfunction resulting in a vicious cycle [9]. Neurons have high polyunsaturated fatty acid content in membranes and a high oxygen consumption but weak antioxidant defense. Hence, they are especially vulnerable to ROS that causes oxidative damage to protein, lipid and nucleic acid [10].

Translocase of the outer mitochondrial membrane 40, Tom40, a pore protein subunit of Tom complex of mitochondria, is responsible for facilitating nuclear-encoded protein import into the mitochondrion [11]. Several studies demonstrate the pathological significance and therapeutic interests of Tom40 in the neurodegenerative disease [12]. Tom40 is reported to have decreased mRNA and protein levels in the peripheral blood and frontal cortex of AD subjects [13–15]. In support of this finding, a longitudinal follow-up study of Tom40 gene expression was conducted over a two-year sampling. It revealed a significant reduction of Tom40 expression levels consistently as the disease progressed [16]. In the in vitro and in vivo studies, Tom40 depletion led to an increased ROS content, oxidative DNA damage as well as mtDNA deletions. The studies revealed reduced mitochondrial integrity and ATP production as well [17]. It is noteworthy that Tom40 overexpression resulted in the restoration of mitochondrial function as observed by increased ATP level, improved bioenergetics, and mitochondrial membrane potential. Moreover, the reduction of oxidative burden as observed by decreased ROS and DNA lesions supports a role for Tom40 as a therapeutic target in the neurodegenerative pathology [18]. Hence, the neuroprotective effects of Tom40 can be utilized to design therapy to ameliorate mitochondrial dysfunction and oxidative stress. Utilizing appropriate means for efficient delivery of the therapeutic Tom40 protein is the key factor in this regard.

Exosome-based delivery system has stability, specificity, and safety. When the multivesicular body (MVB), an intermediate endocytic compartment, fuses with the plasma membrane, exosomes are released outside [19]. They are about 40–100 nm nanovesicles originating from MVB as intraluminal vesicles (ILV) [20]. Carrying the blueprint biomolecules from the cells of their origin, the exosomes function in intercellular communication. Moreover, being composed of a lipid membrane instead of synthetic polymers, exosomes are the next-generation drug delivery platform, a mimic of “nature’s delivery system” [21]. Unlike liposome or polymeric nanoparticles, exosomes can directly unload their cargoes while evading endosomal or lysosomal degradation [22]. Additionally, in the context of brain-related disease therapy, exosomes have high utility as they can cross blood-brain barrier (BBB) [23]. Although there are several studies on exosomes mediated siRNA or miRNA delivery to cancer cells, delivery of cargo protein via exosomes as therapeutic agents in neurodegenerative diseases has not been extensively studied [24]. In a recent study, exosome loaded with catalase were given to *in vitro* and *in vivo* PD models to reduce inflammation and neurodegeneration [25].

In this study, we have used engineered exosomes to deliver Tom40 protein to Human embryonic kidney cells (HEK293) to examine its protective effect against hydrogen peroxide-induced oxidative stress. We have recruited SBI’s XPack Lentivector exosome engineering system to pack the protein in the exosomes. We determined not only the protective effect of Tom40 but also the potential of exosomes as drug delivery vehicles to modulate the cellular bioenergetics.

## Methods and materials

### Cell culture

HEK293 cell line was obtained from American Type Culture Collection (ATCC, Manassas, VA, USA) and cultured in DMEM containing 10% (v/v) FBS, 1% (v/v) L-glutamine, 1% (v/v) nonessential amino acids and 1% (v/v) of both penicillin and streptomycin. The cells were grown in an incubator with optimal culture conditions of 37°C and 5% CO_2_, and the medium was routinely replaced every 2–3 days. For every experiment, both control and test exosomes were harvested from the same batch of media preparation.

### Generation of Tom40 stably overexpressing cells

To generate HEK293 cells overexpressing Tom40, human Tom40 cDNA was purchased from GenScript (NM_001128917.2, clone #OHu16384) and cloned into pULTRA lentivector (Addgene plasmid #24129) using conventional molecular techniques. For a control, we used the original pULTRA lentivector caring only GFP for the lentivirus production. Lentiviruses control and carrying target genes were produced in HEK293T cells using Addgene Lentiviral Packaging Mix (pLP1, pLP2 and pLP-VSVG). The viral particles were then transduced in HEK293 cells. The GFP-positive cells were selected using FACS and used for the subsequent experiment.

### Establishment of a cell line stably producing exosome with Tom40

Tom40 was expressed in the exosomes of cells using XPack Exosome Protein Engineering Technology (System Biosciences, SBI). Human Tom40 cDNA was PCR amplified to attach BamHI and EcoRl restriction sites from the donor plasmid (clone #OHu16384, GenScript). This was then cloned into the BamHI and EcoRl sites of CMV-XP-MCS-EF1a-Puro Cloning Lentivector (System Biosciences) to lentivector carrying Tom40 fragment. The amplified Tom40 region was confirmed by Sanger sequencing. Lentiviruses were prepared by transient transfection in HEK293T cells. Transient transfection of HEK293TN cells was done using XPack CMV-XP-MCS-EF1a-Puro Cloning Lentivector carrying Xpack-Tom40 fragment with the addition of Addgene Lentiviral Packaging Mix (pLP1, pLP2 and pLP-VSVG). The viral supernatant was collected, filtered, concentrated using PEG-*it* (cat: LV825A-1, SBI) and centrifuged. For the viral infection of HEK293 cells, viral supernatant and polybrene transduction enhancer (8 μg/mL, cat: TR-1003-G, Millipore Sigma) were added. After 24 h of transduction, Tom40-exosome producing HEK293 cells were selected by puromycin (5μg/ml, ThermoFisher). Exosomes carrying Tom40 protein were collected (referred to as Tom40-exosome) and validated as previously mentioned. As a control, exosomes were isolated from non-transfected HEK293 cells (referred to as Wild Type or WT-exosome).

### Exosome isolation

Exosomes were isolated from conditioned culture media using a modified PEG-NaCl precipitation method [26]. In brief, 20 mL of conditioned culture media was centrifuged at 10,000xg for 30 mins at 4°C. The supernatant was then mixed with 13.3 mL of 20% PEG with 375mM NaCl. The mixture was kept overnight at 4°C. The following day, the tubes were centrifuged at 10000xg for 60 minutes at 4°C. The exosome pellet was re-suspended in 1x PBS (pH 7.4, sans Calcium and Magnesium).

### Characterization of isolated exosomes

#### Immune-labelling of exosomes

To bind the exosomes, coverslips were pre-treated with Poly-L-ornithine solution (1:10 dilution, Sigma Aldrich, P4957) for 24 hours at 37°C. The exosomal samples were allowed to settle and bind to the coated coverslips for 24 hours at 4°C. Samples were then washed three times with PBS and fixed with 4% paraformaldehyde (PFA) for 20 min at room temperature (RT). The exosomal samples were sequentially incubated with primary (Anti-CD9, at dilution 1:500, cat: 312102 Biolegend) and secondary antibodies (Rhodamine TRITC AffiniPure Goat Anti-Mouse IgG H+L, at dilution 1:200, cat:115025003, Jackson Immunoresearch Inc) overnight at 4°C and for 1 hour at RT respectively. Unbound antibodies were removed by washing in PBS and then imaged using Zeiss Axio Observer microscope.

#### Dot blotting

Proteins were extracted from isolated exosomes and subjected to dot blotting using anti-CD9 (sc-13118, SCBT), anti-CD63 (10628D, Thermofisher), and anti-Tom40 (sc-365467, SCBT) antibodies. RIPA buffer (150mM NaCl, 50mM Tris HCl pH8.0, 0.5% sodium deoxycholate, 0.1% sodium dodecyl sulphate, 1% Triton X-100, protease phosphatase inhibitor) was used to lyse exosomes suspended in 1x PBS. To activate the PVDF membrane, it was prewet in 100% methanol for 1 min followed by soaking in ddH_2_O for 2 mins and in TBST (Tris Buffered Saline-Tween) for 5 mins. A Whatman filter paper was soaked in TBST and placed above a dry Whatman filter paper on top of some paper towels. The PVDF membrane was placed on top of the filter stack, and the RIPA buffer lysed exosomal sample was spotted within a pre-marked grid. The membrane was then left to dry for 1.5h at RT and then blocked with blocking solution (TBST with 2% low-fat milk powder) for 30 min at RT with agitation. Primary antibodies were diluted in 1% Bovine serum albumin (BSA) in TBST and incubated for 1 h at RT. Secondary antibody (Goat anti-Mouse HRP, 31430, Invitrogen) was diluted in 2% low-fat milk powder in TBST and incubated for 30 mins at RT. We imaged the blot using ChemiDoc MP Imaging System (BioRad).

#### Western blotting

Isolated exosomes were further enriched using CD63 conjugated magnetic beads [Exosome—Human CD63 Isolation, Ref- 10606D, Invitrogen], following the manufacturer’s protocol. The samples were then lysed using 1X RIPA buffer and 30μg of total protein was loaded into each well for western blotting. Next, we characterized exosomes using antibodies to exosome-specific marker CD9 (sc-13118, SCBT). The presence of Tom40 in the Tom40-exosome was detected using anti-Tom40 (sc-365467, SCBT) antibody. The secondary antibodies used were Goat anti-Mouse IgG (H+L), Secondary Antibody, HRP (31430, Invitrogen). We imaged the blot using ChemiDoc MP Imaging System (BioRad).

### Labeling of exosomes for uptake analysis

According to the manufacturer’s protocol, the exosomes were fluorescently labeled with Vybrant™ DiO Cell-Labeling Solution (cat: V22886, ThermoFisher). Labeled exosomes were purified using Exosome Spin Column MW3000 (Invitrogen™ 4484449) to remove unincorporated dye from reactions. To examine the uptake of exosomes by cells, 1×10^4^ cells were treated with 10 μg labeled exosomes for 8, 16 and 24 h. After incubation, cells were washed with 1xPBS three times. Cells were then fixed with 4% PFA and stained with DAPI (4′,6-diamidino-2-phenylindole, SouthernBiotech cat: 0100–20). Imaging was performed using the Zeiss Axio Observer microscope.

### Cell viability and metabolic assay

The viability of HEK293 cells was determined using an MTT assay. The MTT assay is a colorimetric assay for assessing cell metabolic activity. NAD(P)H-dependent cellular oxidoreductase enzymes can reduce the tetrazolium dye MTT (3-(4,5-dimethylthiazol-2-yl)-2,5-diphenyltetrazolium bromide) to its insoluble formazan, which has a purple color, reflecting the number of viable cells present. In brief, HEK293 cells (1×10^4^ cells/well) were seeded into a 96-well plate. To normalize, we measured the total protein of the exosome samples without lysis using the direct A_280_ method with Nanodrop 8000 spectrophotometer (Thermofisher) according to the manufacturer’s instructions. Briefly, we applied 2μl of well-resuspended exosome solution between the two optical fibers of the Nanodrop 8000 spectrophotometer and measured absorbance at 280nm. This method has been used by other researchers to normalize the number of exosomes using a minimum volume of sample [27–29]. For treatment of HEK293 cells, we added 10μg total protein of either WT-exosomes or Tom40-exosomes per well. After 16h of treatment, the cells were exposed to 250, 500, 750, 1000, 1250, or 1500 μM Hydrogen peroxide (H_2_O_2_) for 4 h. Following the treatment, 10μl MTT (5 mg/mL, Sigma Aldrich cat: M5655) was added to each well (100μl of media). After 3 h of incubation at 37°C, the medium containing MTT was replaced with 100μl of DMSO to dissolve the purple MTT formazan. Absorbances (Abs.) were read at λ = 570nm by a microplate reader. Cell viability was expressed as a percentage of viable cells in the treated groups compared to the untreated control group.


$$Cell\;viability\;(\%)=\frac{Abs.(sample)-Abs.(blank)}{Abs.(control)-Abs.(blank)}X\;100$$

### Quantitative Real-Time PCR

Total RNAs from HEK293 cells treated with either WT-exosomes or Tom40-exosomes were extracted using Direct-Zol RNA kit (Zymoresearch) and 4μg of total RNA was transcribed into cDNA with SuperScript™ IV Reverse Transcriptase (Invitrogen). The cDNAs were amplified as templates with the random hexamer primers, and Real-Time quantitative PCRs were carried out with Applied Biosystems™ QuantStudio™ 7 Flex Real-Time PCR System (Thermofisher). The transcript abundance relative to WT-exosomes treatment was analyzed by the 2^–ΔΔCt^ method, and β-actin was utilized as an internal gene for normalizing. Primers used in the experiments are listed in S1 Table.

### Statistics

We used the student t-test for western blot and RT-qPCR analysis. We used two-way ANOVA for the cell viability assays. Graphs were generated using Graphpad Prism 9 and the significance level was corrected for multiple testing by false-discovery rate (PFDR < 0.05).

## Results

### Tom40 protects against hydrogen peroxide-induced oxidative stress

**Fig 1:**
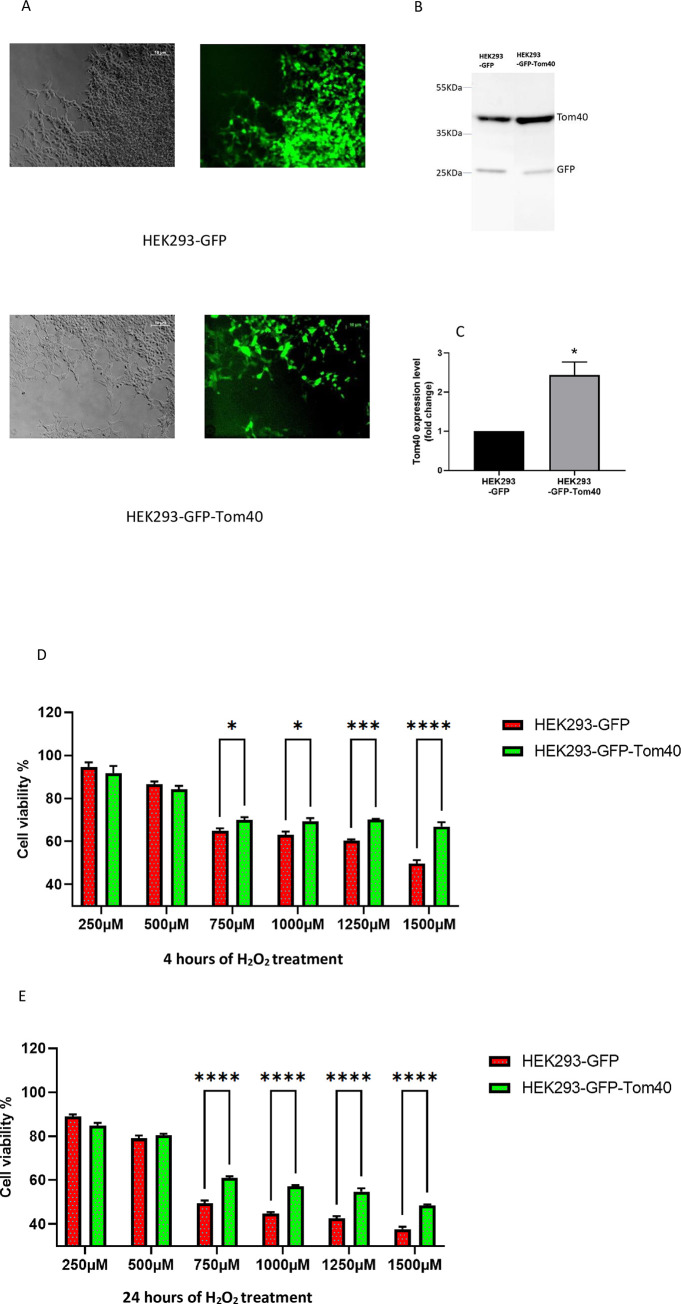
Tom40 protects against hydrogen peroxide-induced oxidative stress. (A) HEK293 cells overexpressing Tom40, or null vector were isolated using FACS through GFP signal. The left panel shows a bright-field image, while the right panel shows GFP fluorescence imaged with a fluorescence microscope. (B) Detection of Tom40 using western blot analysis. The band intensity was measured using ImageJ software and normalized by GFP band signals followed by graphical representation. Student t-test was performed (*P<0.05). (C) Cell viability was performed after 4 hours of H_2_O_2_ treatment using MTT assay. (D) Cell viability was measured after 24 hours of H_2_O_2_ treatment using MTT assay. The data are means ±SEM of 5 replicates and analyzed by Two-way ANOVA. The significance level was corrected for multiple testing by false-discovery rate (*p < 0.05, ***p<0.001).

To determine whether Tom40 overexpression exerts cellular protection, we generated a stable HEK293 cell line overexpressing transgene Tom40 (**Fig 1A**). Expression levels were quantified using western blot analysis (**Fig 1B**). HEK293 cells (HEK293-GFP-Tom40) expressed around 2.4-fold more Tom40 protein than that in the cells stably transduced with null vector (HEK293-GFP) (**Fig 1C**). To induce oxidative stress, we used varying concentrations of hydrogen peroxide (H_2_O_2_) for a short period (4 hours) and a long period (24 hours). The cellular viability was evaluated by MTT method. Under the short period of induced oxidative stress, Tom40 overexpressing cells showed significant protection at 750μM, 1000μM, 1250μM, and 1500μM H_2_O_2_ treatment (**Fig 1D**). Most protection, about 17.16%, compared to control was observed at 1500μM. For a longer period of induced oxidative stress, Tom40 overexpression continued to give significant cellular protection (10–12% more) than control (**Fig 1E**). These findings indicate that overexpression of Tom40 significantly protected cells from oxidative injury.

### Exosome characterization and cellular uptake

**Fig 2:**
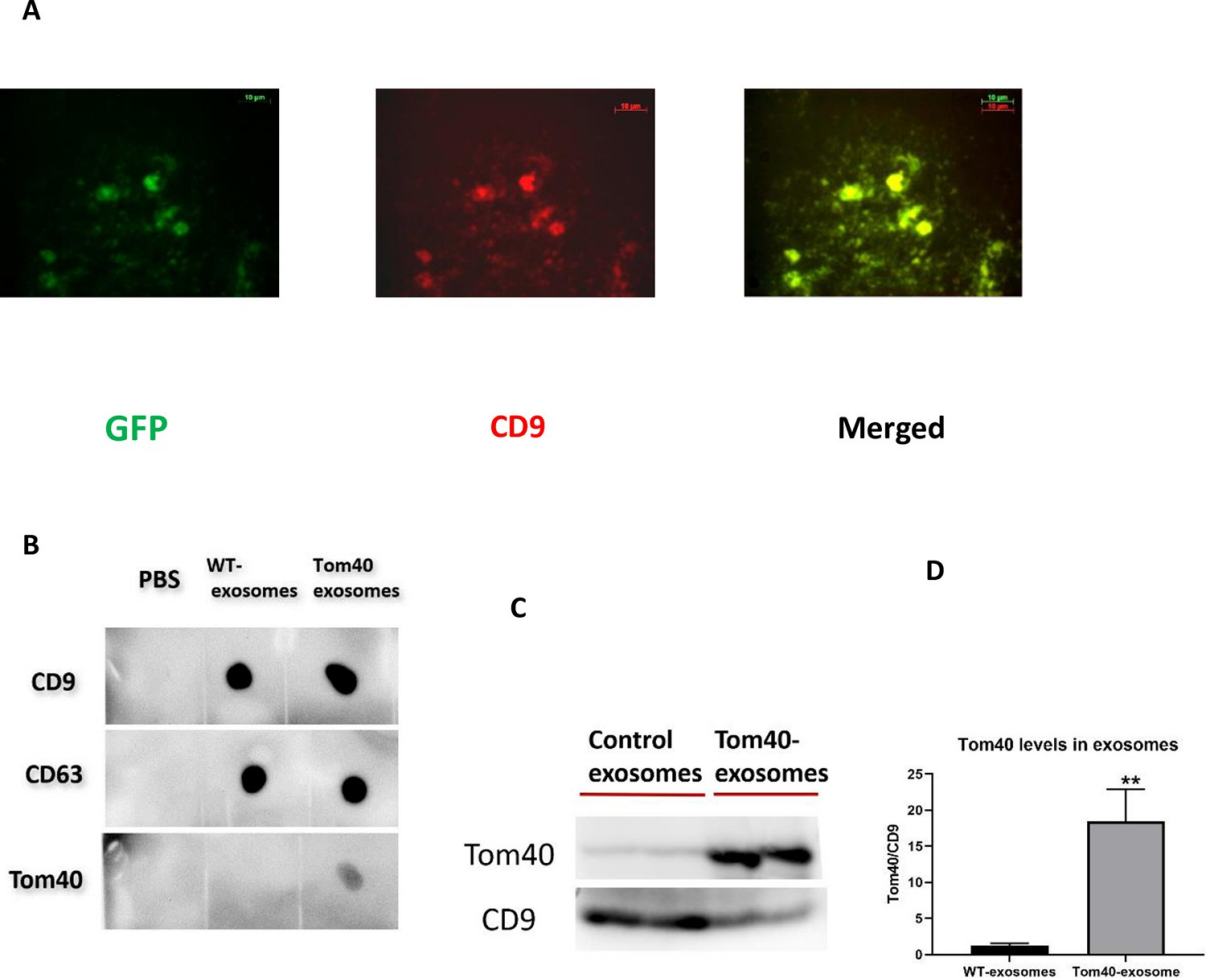
Exosome characterization. (A) Immunocytochemical detection of CD9 as an exosomal marker on GFP-packed exosome. Imaged with Fluorescence microscope at 400X total magnification. (B) Dot blot analysis of isolated exosomes for CD9 and CD63 as exosome markers. Detection of Tom40 signal for Tom40-exosome. PBS as a negative control. (C) The significant presence of Tom40 protein in isolated Tom40-exosome was shown in Western blot analysis. (D) Graphical representation of Tom40 signal normalized by CD9. Student t-test was performed *P<0.05.

Exosomes were isolated from the conditioned media of HEK293 cells by using a modified PEG-NaCl precipitation method [26]. To determine the efficacy of the exosome isolation method, we used XPack-GFP lentivector (SBI, Cat # XPAK530PA-1) to generate stable HEK293 cells constitutively producing GFP-packed exosomes. XPack peptide sequence targets the protein of interest, in this case GFP, to the interior exosomal membrane. The GFP-packed exosomes were isolated using the modified precipitation method and characterized in immunostaining analysis (**Fig 2A**). We confirmed CD9 as an exosomal marker and the presence of GFP packed in the exosomes. To produce Tom40 protein-containing exosome, we cloned human Tom40 cDNA into SBI’s XPAK-510-PA-1 vector. This allows Tom40 to be packed into exosomes for secretion. We performed Dot blot analysis to validate the exosomal presence. Along with CD9, CD63 is another commonly used exosomal marker. Dot blot revealed the presence of CD9 and CD63 for both WT-exosomes and Tom40-exosomes (**Fig 2B**). However, only Tom40-exosomes showed a positive for Tom40 immunoreactivity. For further validation, we enriched the exosomes with anti-CD63 conjugated magnetic beads and used them as templates in Western blot analysis (**Fig 2C and 2D**). Tom40-exosome had significantly higher levels of Tom40 protein as compared to wild-type control exosomes.

**Fig 3:**
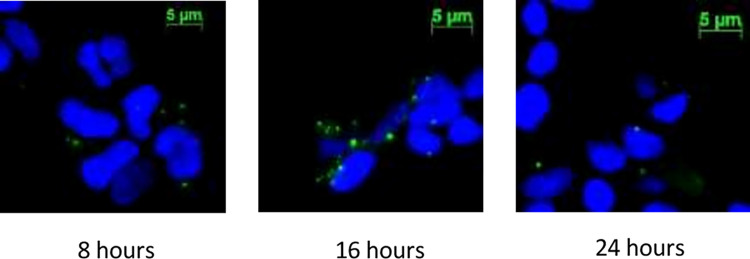
Cellular uptake analysis. HEK293 cells were incubated with DiO stained Tom40-exosome for 8, 16 and 24 hours. Imaged with Fluorescence microscope at 400X total magnification. DiO stained Tom40-exosomes were seen as green fluorescent staining. All nuclei were counterstained with DAPI (blue).

To examine the cellular uptake of Tom40-exosomes, Tom40-exosomes were stained with green lipophilic tracer dye DiO. HEK293 cells were then incubated with DiO-stained Tom40-exosomes for 8, 16 and 24 hours (**Fig 3**). After incubation, the cells were washed adequately by PBS to get rid of any undesired DiO signal. We observed that the exosomes were taken up by cells as early as 4 hours. However, at around 16 hours we observed the most uptake of exosomes. Cellular uptake slowed down at 24 hours.

### Exosome mediated Tom40 delivery protects cells from oxidative stress

**Fig 4:**
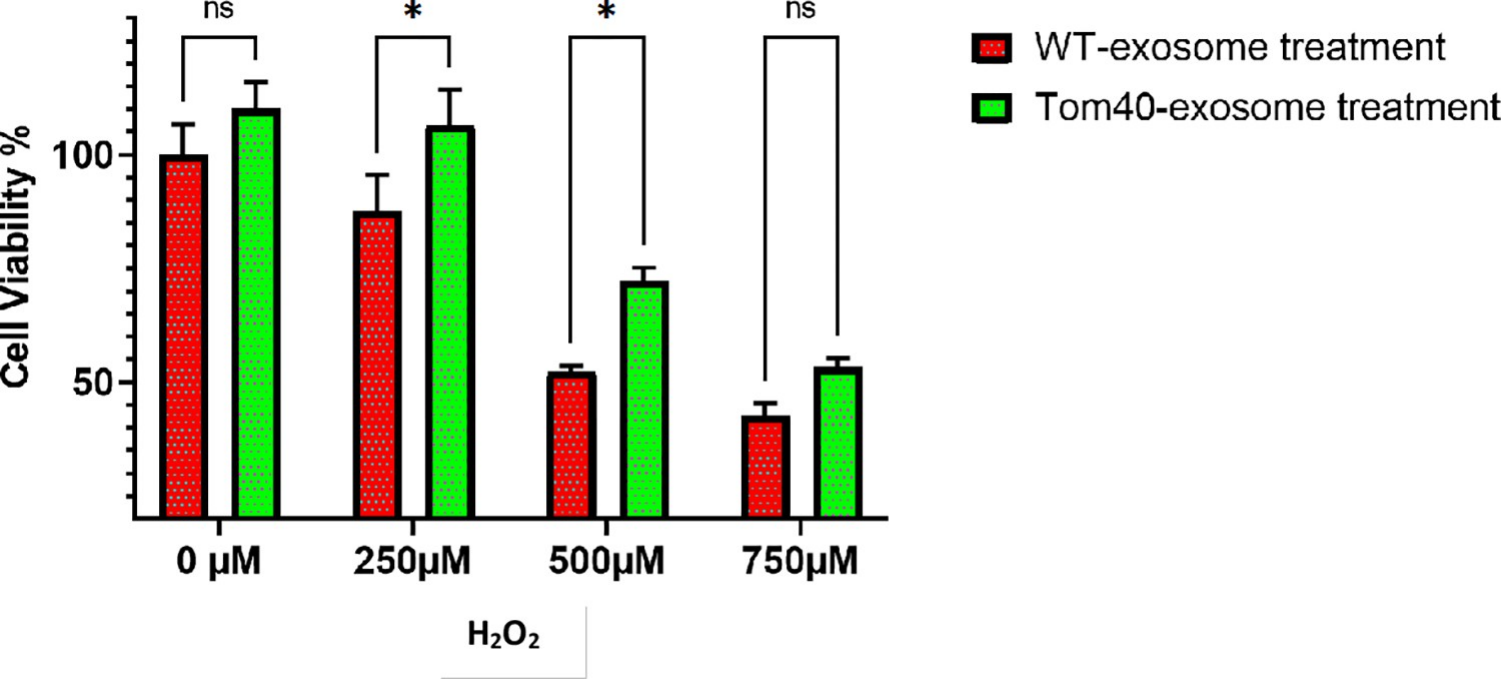
Tom40-exosome protects HEK293 cells against H_2_O_2_ induced oxidative stress. Cells were incubated with exosomes for 16 hours and then treated with H_2_O_2_ for 4 hours. MTT assay was performed to detect metabolically viable cells. The data are means ±SEM of 4 replicates. The data were analyzed by Two-way ANOVA and the significance level was corrected for multiple testing by false-discovery rate (*P < 0.05.).

To determine the protective effects exerted by exosome-mediated delivery of Tom40 against oxidative stress, we incubated HEK293 cells with either Tom40-exosome or WT-exosomes. After 16 hours of incubation, we treated the cells with varying concentrations of H_2_O_2_ for 4 hours. Significant cellular protection was observed at 250μM and 500μM H_2_O_2_ treatment (**Fig 4**).

### Exosome mediated Tom40 delivery regulates gene expression related to mitochondrial function

**Fig 5:**
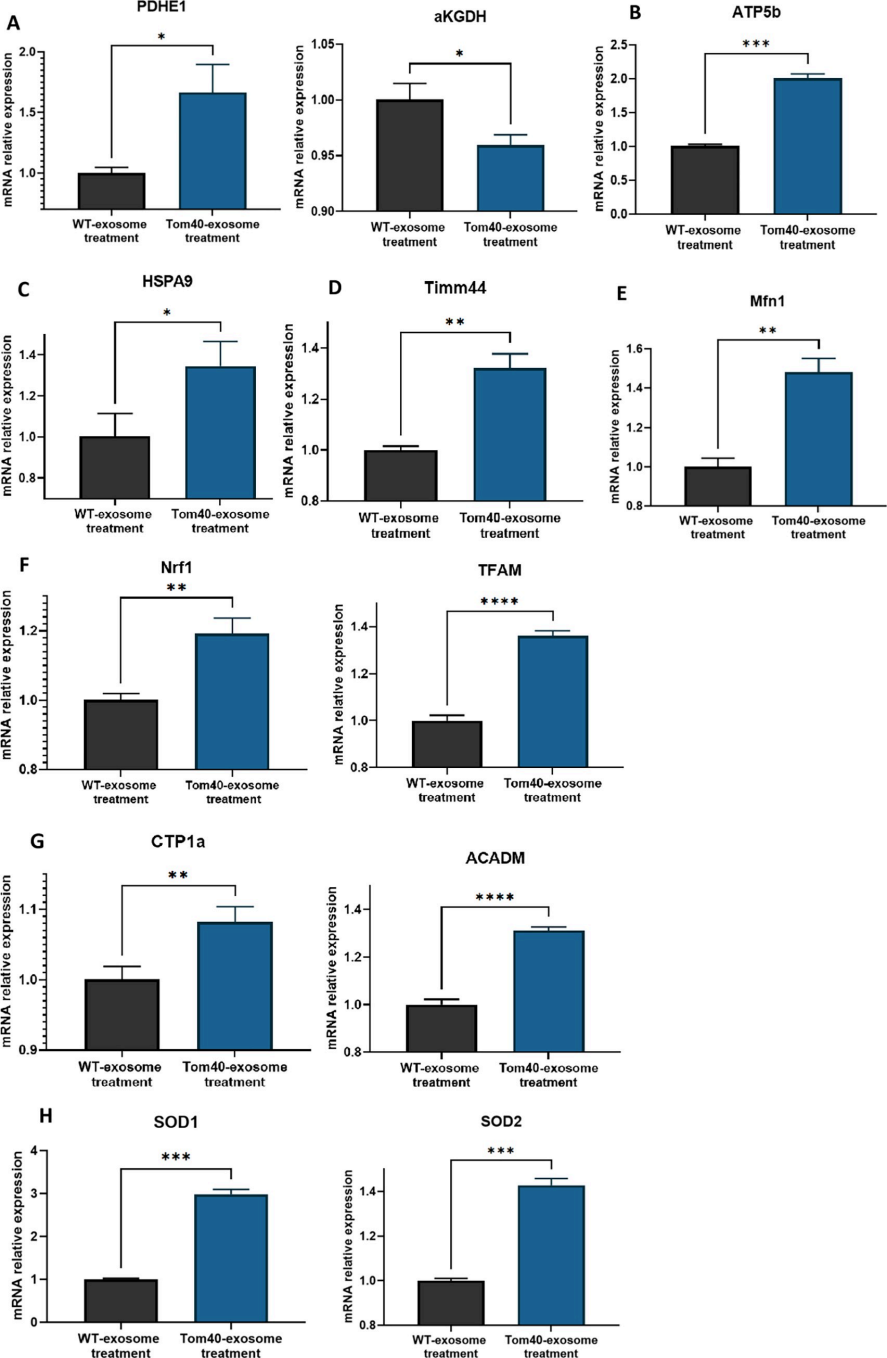
Tom40-exosome regulates mitochondrial functional genes. Relative expression levels of genes relevant to mitochondrial function using RT-qPCR. (A, B) Expression levels of genes involved in mitochondrial bioenergetics, (C) Expression levels of genes encoding for molecular chaperone, (D, E) Expression levels of genes involved in mitochondrial membrane proteins, (F) Expression levels of genes involved in mitochondrial transcription (G) Expression levels of genes involved in mitochondrial fatty acid oxidation, (H) Expression levels of genes involved in anti-oxidant system. The asterisk indicates a significant difference (Student’s t-test performed, ∗P < 0.05, ∗∗P < 0.01, ***P<0.001).

Previous research works have demonstrated that changes in Tom40 expression modulate mitochondrial bioenergetics [18]. It is also reported that Tom40 overexpression is associated with increased ATP formation [30]. To determine if Tom40 protein delivery through exosome changes the expression of genes related to mitochondrial bioenergetics, we incubated HEK293 cells with Tom40-exosome for 16 hours and checked gene expression levels using quantitative real-time PCR. We observed that genes involved in mitochondrial bioenergetics, PDHE1α (Pyruvate dehydrogenase E1 component subunit alpha) and αKGDH (α-ketoglutarate dehydrogenase), had altered expression upon Tom40-exosome delivery compared to control (**Fig 5A**). We also inferred increased ATP formation as observed in increased ATP5b (ATP synthase F1 subunit beta) level (**Fig 5B**).

The mitochondrial chaperone is found at a higher level upon Tom40 overexpression through the gene delivery [18]. In the current study, the expression data showed a significant increase in mitochondrial chaperone gene HSPA9 (mt-HSP70) upon Tom40-exosome incubation compared to WT-exosomes (**Fig 5C**). We also checked the expression of mitochondrial membrane proteins, Timm44 (Translocase of Inner Mitochondrial Membrane 44) and Mfn1 (Mitofusin1). Significant upregulation for both the genes was observed (**Fig 5D and 5E**).

We further investigated whether exosome-mediated Tom40 delivery would affect the genes involved in mitochondrial transcription, Nrf1 (Nuclear respiratory factor 1) and TFAM (Transcription Factor A, Mitochondrial). Both Nrf1 and TFAM were upregulated in Tom40-exosome treated HEK293 cells (**Fig 5F**). Tom40-exosome treatment of HEK293 cells was also able to regulate genes involved in mitochondrial fatty acid oxidation. Carnitine palmitoyl transferase 1A (CPT1α) and Medium-chain acyl-CoA dehydrogenase (ACADM) are two key genes (**Fig 5G**). We observed upregulation of CPT1α and ACADM in Tom40-exosome treated HEK293 cells compared to Control.

We were interested to see whether Tom40 could affect superoxide dismutase (SOD) expression. SODs are key enzymes in oxidative stress resistance reactions [31]. SOD1 and SOD2, both antioxidant genes were upregulated with the delivery of Tom40-exosome (**Fig 5H**). These data indicate that exosome-mediated Tom40 delivery regulates gene expression related to mitochondrial function and general health.

## Discussion

Tom40 is essential for life and causes growth retardation of organisms when knocked down [32–35]. As mentioned earlier, in neurodegenerative diseases like AD and PD, Tom40 is reported to have reduced mRNA and protein expression [13–15]. The previous finding suggests that Tom40 overexpression could provide a protective benefit for cells against oxidative damage [17]. Another study showed overexpression of Tom40 increased expression levels of crucial mitochondrial protein in mitochondrial protein quality control (Mt-PQC) [18] These findings suggest that Tom40 is potentially a remunerative agent in clinical therapy of neurodegenerative diseases. Because exosomes are clinically safe and generally non-immunogenic, our present study involves exosome as a drug delivery vehicle to investigate the effects of delivery of Tom40 on recipient cells [21]. The study also investigates the potential tolerance of the recipient cell to oxidative stress. We have shown that the exosome-mediated delivery of Tom40 protein gives significant protection to cells against H_2_O_2_ -induced oxidative damage. Tom40 delivery through the exosome also upregulates key genes involved in mitochondrial homeostasis. Our data indicate that Tom40 delivery using exosomes may have a key role in clinical therapy to alleviate oxidative stress in neurodegenerative diseases.

Tom40 overexpressing cells were previously reported to have resisted the cytotoxic effect of Aβ_1−42_ added to the medium [18]. Aβ_1−42_ possesses the ability to induce cytotoxicity through oxidative stress [36]. In our study, we generated a stable cell line overexpressing Tom40 and induced oxidative stress by H_2_O_2_. We observed significant cellular protection exerted by Tom40 overexpressing cells for both short and long periods of time in the MTT assay. MTT assay determines the number of metabolically active cells by measuring the amount of cellular oxidoreductase enzyme [37]. Our data indicate that Tom40 may have rendered protection through improving cellular bioenergetics.

We used a modified PEG-NaCl method established in our lab to isolate the exosomes [26]. The presence of exosomal markers was determined using dot and western blot analysis. We observed that at around 16 hours of incubation most of HEK293 cells uptook exosomes. This confirms that the cellular uptake of exosomes is time-dependent. We performed the viability assay and subsequent gene expression study with 16 hours of exosome incubation. We engineered exosomes using SBI’s XPack lentivector exosome technology. Tom40 protein was packed successfully inside the exosome in the transfected cell line. Tom40 delivery through exosome exerted cellular protection against H_2_O_2_ induced oxidative stress. We used lentivirus to make a stable HEK293 cell line constitutively overexpressing Tom40. In Fig 1C, Western blot showed that HEK293 cells received Tom40 lentivirus had 2.4-fold Tom40 expression compared to the control cells treated with null lentivirus. On the other hand, HEK293 cells treated with Tom40 exosome treatment delivery showed a 15-20-fold increase in Tom40 level compared to those received WT-exosome (Fig 2D). These results indicate that the cells that received more Tom40 got better protection against oxidative stress.

Most age-related neurodegenerative diseases have reduced bioenergetics. Mitochondrial dysfunction is directly associated with altered activities of key mitochondrial enzymes, pyruvate dehydrogenase and α-ketoglutarate dehydrogenase complexes [38]. In a previous study, Tom40 overexpression increased the levels of PDHE1α and αKGDH [18]. Our findings show that exosome-mediated Tom40 delivery regulated the expression level of both these enzymes. PDHE1α is a subunit of pyruvate dehydrogenase complex that converts pyruvate into acetyl-CoA. An increase in PDHE1α expression may have therapeutic benefits for age-related diseases [38]. αKGDH is a highly regulated enzyme that determines the metabolic flux of Krebs cycle [39]. In our study, PDHE1α expression was upregulated significantly while αKGDH was downregulated a little. The αKGDH level was likely decreased as compensation to the increased PDHE1α expression to maintain the overall bioenergetics. Decreased ATP formation is also associated with the neurodegeneration [40]. In the current data, we observed significant upregulation of ATP5b with Tom40-exosome treatment. ATP5b is a subunit of ATP synthase that catalyzes ATP [41].

HSPA9 (or Mortalin, Grp75, PBP74, mtHsp70) is one of the key mitochondrial chaperones which plays important role in dealing with oxidative stress in degenerative neurons [42]. In a previous study, HSPA9 was shown to increase in level upon Tom40 overexpression [18]. The present data also demonstrates the increase in expression after incubation with Tom40 containing exosome. HSPA9 could be a potential target for therapies based on oxidative stress. It is reported that increased expression of HSPA9 confers axonal protection and modulates mitochondrial dynamics in neurons [43]. HSPA9 interacts with Timm44 to maintain the mitochondrial protein quality control mechanism [44]. Timm44 gene delivery reduces mitochondrial superoxide production and facilitates the import of antioxidative enzymes [45]. In the present study, we observed increased Timm44 expression upon exosome-mediated Tom40 delivery. Like Timm44, which is a membrane protein on the inner membrane, Mfn1 is another membrane protein that resides on the outer membrane that play role in the mitochondrial fusion [46]. In neurodegenerative diseases, mitochondrial fusion proteins are downregulated [47]. In this report, we found increased expression of Mfn1 upon exosome-mediated delivery of Tom40.

An efficient cellular response against oxidative stress activates the transcription of various antioxidant genes through cis-acting antioxidant response element (ARE). Such transcription factor, Nrf1, binds to ARE and induces transcription of enzymes that catalyse glutathione synthesis, a major antioxidant [48]. Another transcription factor, TFAM, is also crucial. TFAM expression is reported to improve cognitive function, render protection against oxidative stress, and maintain the mitochondrial organelle genome [49–51]. In our current data, Tom40-exosome treatment upregulated both Nrf1 and TFAM expression compared to control exosome treatment. Upregulation of these mitochondrial transcription factors may be therapeutically useful in neurodegenerative diseases where mitochondrial dysfunction and oxidative stress are major pathologies.

We also explored the ability of the Tom40-exosome to regulate gene expression related to mitochondrial fatty acid oxidation. CPT1α is a key regulator of lipid metabolism. Memory deficits in the AD mice model were reversed through an increased CPT1a expression [52]. ACADM deficiency also leads to defective Mt-PQC [53]. We observed increased expression of CPT1a and ACADM upon Tom40 delivery through the exosome. This is of significance as disruption of these enzymes often leads to mitochondrial dysfunction [54].

Superoxide dismutases (SODs) are major players in defending cells against oxidative stress [55]. The dismutase activity converts the superoxide anions to H_2_O_2_ and oxygen. Thereby, SODs help in reducing reactive oxygen species. Both SOD1 and SOD2 play an active role against oxidants, SOD1 (Cu/Zn SOD) is widely reported as a cytosolic isoform while SOD2 (Mn-SOD) is associated with the mitochondria [56]. The SODs can also act as transcription factors and activate genes related to the oxidative stress resistance [56–58]. We analyzed SOD1 because it is found in not only the nucleus, lysosomes, and peroxisome but also in the intermembrane space (IMS) of mitochondria [56, 57]. It may play a role in removing superoxide released from the mitochondrial respiratory chain. It is reported that SOD1 overexpression alters ROS production and reduces neurotoxic inflammatory signalling as well [58]. In the current findings, both SOD1 and SOD2 were upregulated in Tom40-exosome treated HEK293 cells compared to WT-exosomes. This data indicates the potential of Tom40 in activating oxidative stress resistance genes.

Here we report that Tom40 protects cells from oxidative stress by viral induction and exosomal delivery. In neurodegenerative diseases including AD, Tom40 is reduced at transcriptional and protein levels [13–15]. It is possible that due to the reduced level of Tom40 in mitochondria, the nuclear-encoded mitochondria-targeted proteins fail to enter mitochondria, thereby causing degradation. Hence, Tom40 delivery through the exosome may help compensate for the low levels of Tom40 in AD. In our study, exosome-mediated Tom40 delivery protected the cells from oxidative stress generated by H_2_O_2_ and found upregulated mitochondrial and SODs gene transcriptions. Tom40 facilitates protein entry to mitochondria and is not likely to directly affect these gene expressions. Cells maintain mitochondrial function using regulatory mechanisms of both the nucleus and mitochondria and dysfunction of the crosstalk between these two systems may lead to dysregulation of cellular pathways and degeneration [59, 60]. Thus, enhanced protein trafficking in mitochondria by exosomal delivery of Tom40 may have signaled transcription of the target genes as a secondary effect. The increased expression of cytosolic SOD1 and mitochondrial SOD2 along with the target genes that are involved in the antioxidant response in the cells may play a role in Tom40 protection against cytoplasmic and mitochondrial oxidative stress in general. Although these findings may indicate the protective function of Tom40, further investigations on the protein localization and trafficking are needed to determine the mechanism of action. Our key finding in this study is to demonstrate the protective function of Tom40 against oxidative stress using exosomes as the delivery vehicle. Exosome-mediated drug delivery system could be a potential clinical tool to deliver functional protein, such as Tom40, for neurodegenerative disease therapy.

## Supporting information

S1 Table List of primers. These primer sets were used for quantitative real-time PCR to check gene expression used in Fig 5.
(DOCX)

S1 Raw images Original raw image data. Raw original images for microscopy in Fig 1A (A), the Western blot analysis performed in Fig 1B (B), Immunocytochemistry of CD9 as exosomal marker on GFP-packed exosome in Fig 2A (C), the dot blot analysis performed in Fig 2B (D), the western blot analysis performed in Fig 2C (E) and cellular uptake analysis performed in Fig 3 (F).
(PDF)

S1 File Minimum underlying data. Minimum underlying data for each figure.
(DOCX)
