# Supplementary material for: Exosome mediated Tom40 delivery protects against hydrogen peroxide-induced oxidative stress by regulating mitochondrial function
Source: PLoS One. 2022 Aug 11;17(8):e0272511. doi: 10.1371/journal.pone.0272511 (PMC9371349; doi:10.1371/journal.pone.0272511)
Supplement: S1 Table — These primer sets were used for quantitative real-time PCR to check gene expression used in Fig 5. (DOCX) [file pone.0272511.s001.docx]

**S1Table.**

| Gene | Sense | Sequence 5'-3' |
| --- | --- | --- |
| PDHE1α | forward | CGGGCTCACGGCTTTACTTTCA |
|  | reverse | ATGCCATTGCCCCCGTAGAAGT |
| αKGDH | forward | ATGCTGATCTGGACTCCTCCGT |
|  | reverse | TGAAAGTGGTGGTGGGCAAGTG |
| ATP5b | forward | AGGTGTCTGCATTATTGGGCCG |
|  | reverse | TAGTAGCAGGGGCAGGGTCAGT |
| HSPA9 | forward | TACTACCAACTCCTGCGTGGCA |
|  | reverse | TGGTTCTGGCACCTTCGGCATT |
| Timm44 | forward | GGCCGAGTCGGTATCCAAAG |
|  | reverse | CTCCCGCAAACTCCGTTCTC |
| Mfn1 | forward | GATGCACCGATGAAGTAAACG |
|  | reverse | GCCCAGGGAAAAACGAAATACAA |
| Nrf1 | forward | TGGAACAGCAGTGGCAAGATCTCA |
|  | reverse | GGCACTGTACAGGATTTCACTTGC |
| TFAM | forward | GCGCTCCCCCTTCAGTTTTG |
|  | reverse | GTTTTTGCATCTGGGTTCTGAGC |
| qCTP1a | forward | TTATCGTGGTGGTGGGCGTGA |
|  | reverse | ACGCCGCTGACCACGTTCTT |
| ACADM | forward | AACGGGAGCCAACATGGCAG |
|  | reverse | CTGGTTCACGTTGTCGATTGGCT |
| qSOD1 | forward | TTGCATCATTGGCCGCACAC |
|  | reverse | CAAGCCAAACGACTTCCAGCG |
| qSOD2 | forward | TCAGGTTGGGGTTGGCTTGGT |
|  | reverse | CGTGCTCCCACACATCAATCCC |
