# Supplementary material for: Exosome mediated Tom40 delivery protects against hydrogen peroxide-induced oxidative stress by regulating mitochondrial function
Source: PLoS One. 2022 Aug 11;17(8):e0272511. doi: 10.1371/journal.pone.0272511 (PMC9371349; doi:10.1371/journal.pone.0272511)
Supplement: S1 Raw images — Raw original images for microscopy in Fig 1A (A), the Western blot analysis performed in Fig 1B (B), Immunocytochemistry of CD9 as exosomal marker on GFP-packed exosome in Fig 2A (C), the dot blot analysis performed in Fig 2B (D), the western blot analysis performed in Fig 2C (E) and cellular uptake analysis performed in Fig 3 (F). (PDF) [file pone.0272511.s002.pdf]

HEK293-GFP cells

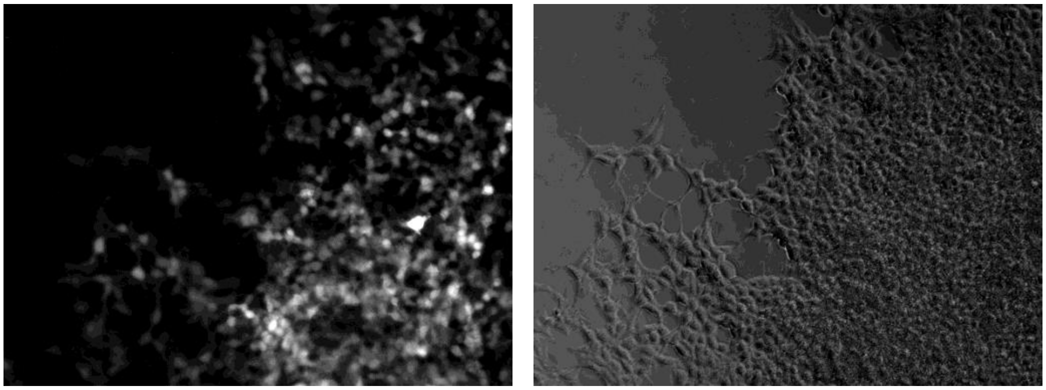

GFP positive cells | Bright field

HEK293-GFP-Tom40 cells

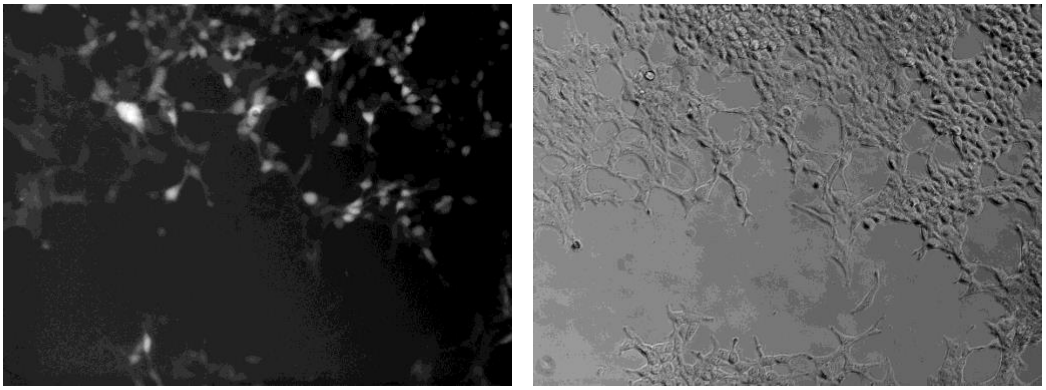

GFP positive cells | Bright field

A. Original raw image of Fig 1A.

HEK293 cells overexpressing Tom40, or null vector were isolated using FACS through GFP signal. The left panel shows GFP fluorescence imaged with a fluorescence microscope, while the right panel shows the bright-field image. GFP-positive cells were imaged using Zeiss Axio Observer microscope. Total 100x magnification.

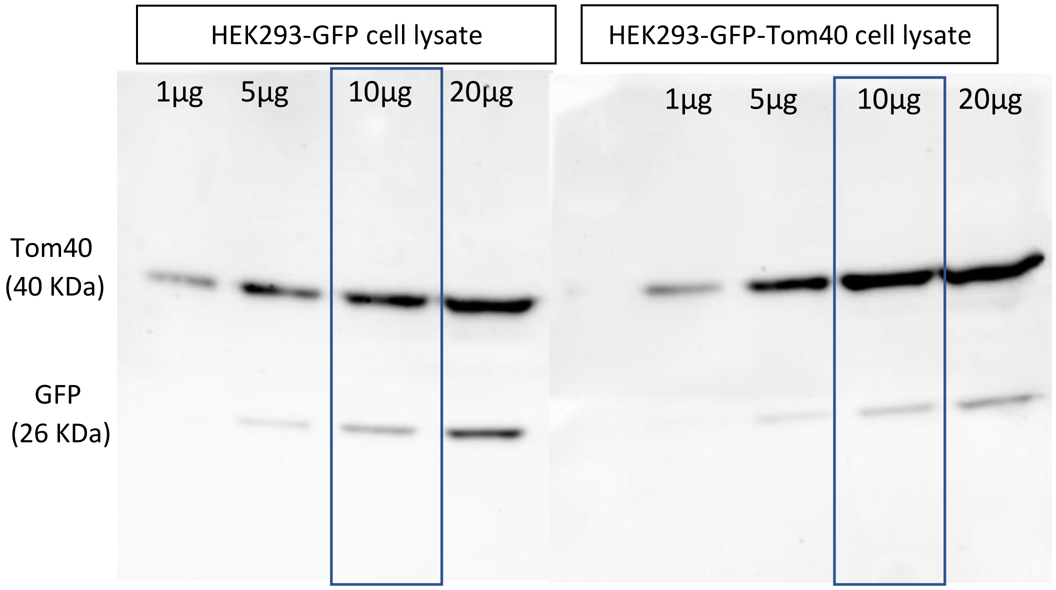

B. Original blot image of the Western blot analysis performed in Fig 1B.

Varying amounts of Total cell lysate for each of HEK293-GFP or HEK293-GFP-Tom40 cells were loaded in the same SDS-PAGE gels. Both HEK293-GFP and HEK293-GFP-Tom40 cell lysate samples were loaded on the same precast NuPage 4-12% bis-tris protein gels cassette. Blotting membrane was cut into 4 pieces for incubations with antibodies recognizingTom40 or GFP. The rectangular panels annotated on each of the blot was used to generate Fig 1B in the manuscript. Each Tom40 band was normalized by the intensity of the GFP band on the same lane. The fold difference between HEK293-GFP and HEK293-GFP-Tom40 for each lysate amount was quantified and plotted on the Fig 1C graph. The image was captured with Chemidoc MP Imaging system (BioRad) and the band intensities were measured using ImageJ editing software.

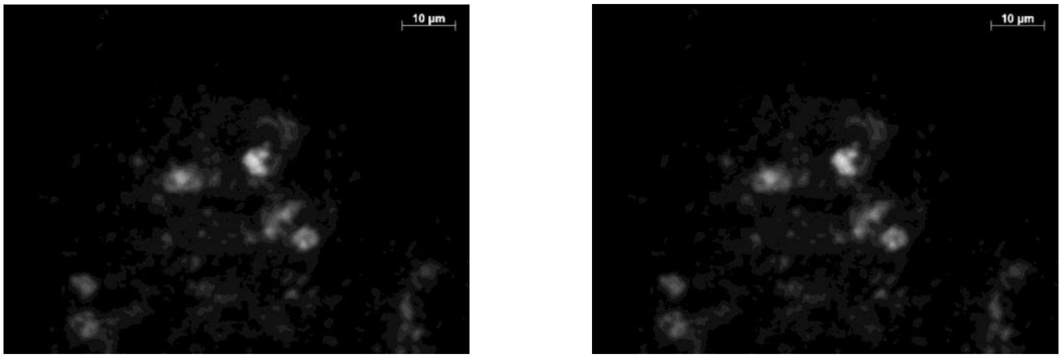

Imaged for GFP

Imaged for TRITC (CD9)

C. Original image of Immunocytochemistry of CD9 as exosomal marker on GFP-packed exosome in Fig 2A

TRITC conjugated anti-CD9 antibody used to stain exosomes. Imaged using Zeiss Axio Observer microscope at total 400x magnification.

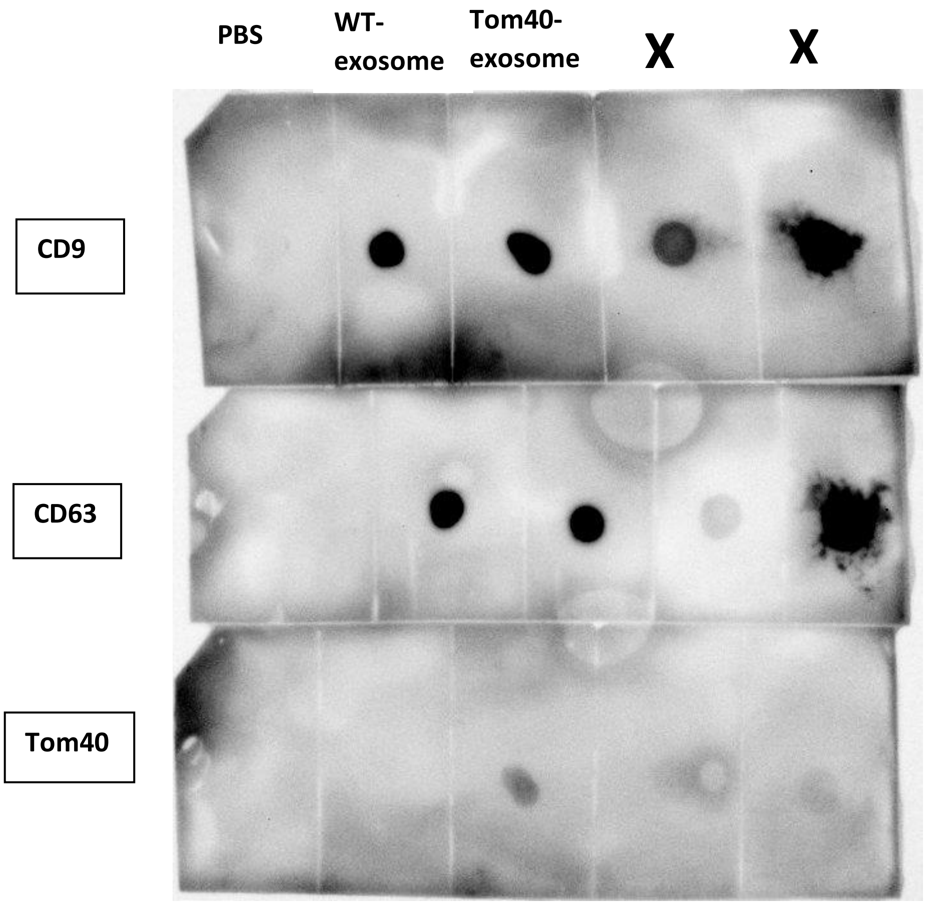

D. Original blot image of the Dot blot analysis performed in Fig 2B

Dot blot analysis of isolated exosome for CD9 and CD63 as exosome markers. Detection of Tom40 signal for Tom40-exosome. PBS as a negative control. The last two columns of the PVDF strips (labelled as 'X') are not part of the experiment. The image was captured with Chemidoc MP Imaging system (BioRad)

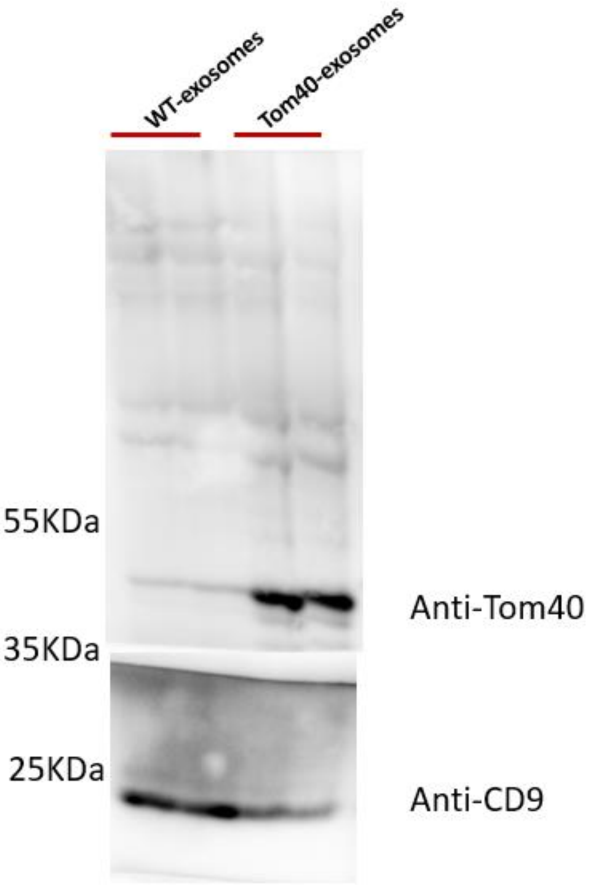

E. Original blot image of the western blot analysis performed in Fig 2C.

The image contains non-specific bands in the lanes. Tom40 is observed around 40 KDa. The molecular weight for CD9 is often observed at 23-27 KDa. The image was captured with Chemidoc MP Imaging system (BioRad) and analyzed using ImageJ editing software.

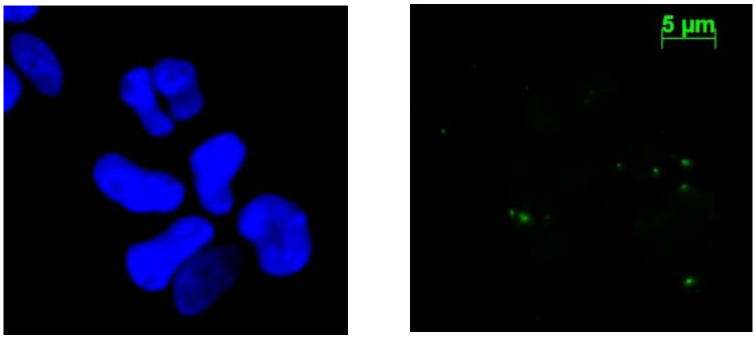

8 hours of incubation

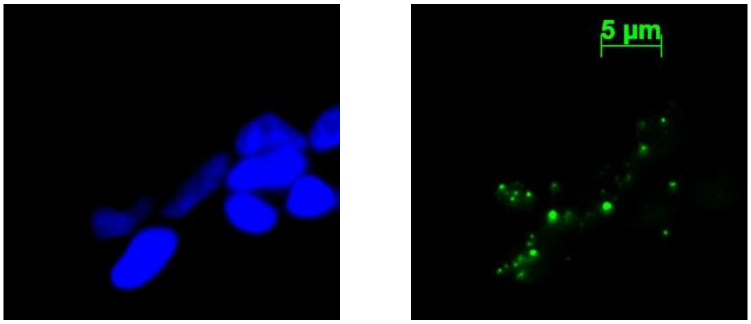

16 hours of incubation

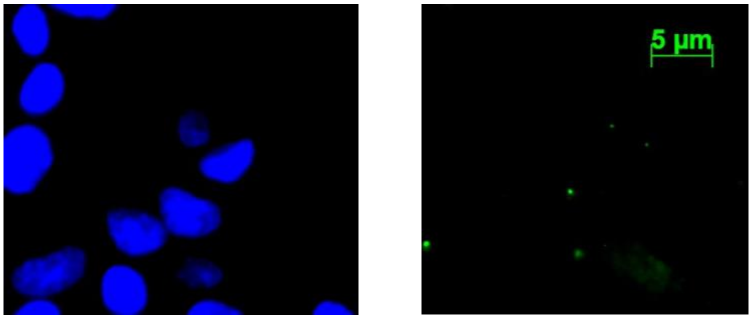

24 hours of incubation

F. Raw image of cellular uptake analysis performed in Fig 3.

HEK293 cells were incubated with DiO stained Tom40-exosome for 8, 16 and 24 hours. Imaged with Zeiss Axio Observer microscope at 400X total magnification. DiO stained Tom40-exosomes were seen as Green staining. All nuclei were counterstained with DAPI (blue)
