# Supplementary material for: Exosome mediated Tom40 delivery protects against hydrogen peroxide-induced oxidative stress by regulating mitochondrial function
Source: PLoS One. 2022 Aug 11;17(8):e0272511. doi: 10.1371/journal.pone.0272511 (PMC9371349; doi:10.1371/journal.pone.0272511)
Supplement: S1 File — Minimum underlying data for each figure. (DOCX) [file pone.0272511.s003.docx]

**Minimum Data for Fig 1C**

Tom40 band intensity was measured in ImageJ software and normalized by GFP band on the same lane. Normalized Tom40 band for each HEK293-GFP lysate amount was compared with equal amount of HEK293-GFP-Tom40 lysate. Mean fold difference is then calculated and plotted on the graph Fig 1C.

Student t-test was performed, and the difference is significant as per the p-value=0.0181. The data points were plotted on the graph using GraphPad prism with Standard deviation (SD)

|  | Mean fold difference |
| --- | --- |
| HEK293-GFP | 1 |
| HEK293-GFP-Tom40 | 2.434 |
| p-value | 0.0181 |

**Minimum data for Fig 1D,E**

**Cell viability assay:**

Cell viability was expressed as a percentage of viable cells in the treated groups compared to the untreated control group. Cells not treated by H_2_O_2_ were considered as control group. The mean viability percentages were plotted on the graph using GraphPad prism with Standard Error of the Mean (SEM).

**4 hours of H_2_O_2_ treatment**

| N=5 | HEK293-GFP mean viability (%) | SEM | HEK293-GFP-Tom40 mean viability (%) | SEM | p-value |
| --- | --- | --- | --- | --- | --- |
| 250 μM H_2_O_2_ | 94.60308 | 2.079 | 91.71532 | 3.371 | 0.1487 |
| 500 μM H_2_O_2_ | 86.60182 | 1.228 | 84.08759 | 1.774 | 0.1582 |
| 750 μM H_2_O_2_ | 64.82586 | 1.195 | 69.96350 | 1.361 | 0.0299 |
| 1000 μM H_2_O_2_ | 63.06872 | 1.498 | 69.37956 | 1.468 | 0.0123 |
| 1250 μM H_2_O_2_ | 60.24474 | 0.715 | 70.16423 | 0.348 | 0.0002 |
| 1500 μM H_2_O_2_ | 49.77251 | 1.551 | 66.93430 | 1.918 | <0.0001 |

**24 hours of H_2_O_2_ treatment**

| N=5 | HEK293-GFP mean viability (%) | SEM | HEK293-GFP-Tom40 mean viability (%) | SEM | p-value |
| --- | --- | --- | --- | --- | --- |
| 250 μM H_2_O_2_ | 88.88346 | 1.0315 | 84.84766 | 1.251 | 0.0073 |
| 500 μM H_2_O_2_ | 78.97498 | 1.346 | 80.31814 | 0.801 | 0.3558 |
| 750 μM H_2_O_2_ | 49.51799 | 1.235 | 61.01375 | 0.644 | <0.0001 |
| 1000 μM H_2_O_2_ | 44.80780 | 0.598 | 57.10434 | 0.509 | <0.0001 |
| 1250 μM H_2_O_2_ | 42.48932 | 1.101 | 54.75869 | 1.504 | <0.0001 |
| 1500 μM H_2_O_2_ | 37.68151 | 1.0346 | 48.44971 | 0.509 | <0.0001 |

**Minimum data for Fig 2D**

Tom40 band intensity was measured in ImageJ software and normalized by CD9 band on the same lane. Mean fold difference is then calculated and plotted on the graph Fig 2D. Student t-test was performed, and the difference is significant as per the p-value=0.0026. The data points were plotted on the graph using GraphPad prism with Standard deviation (SD).

|  | Mean fold difference |
| --- | --- |
| WT-exosome | 1.25302 |
| Tom40-exosome | 18.4644 |
| p-value | 0.0026 |

**Minimum data for Fig 4**

**Cell viability assay :** Cell viability was expressed as a percentage of viable cells in the treated groups compared to the untreated control group. The mean viability percentages were plotted on the graph using GraphPad prism with Standard Error of the Mean (SEM).

| N=4 | WT-exosome treated HEK293 cell viability mean (%) | SEM | Tom40-exosome treated HEK293 cell viability mean (%) | SEM | p-value |
| --- | --- | --- | --- | --- | --- |
| 0 μM H_2_O_2_ | 99.999 | 6.638 | 110.087 | 5.964 | 0.1905 |
| 250 μM H_2_O_2_ | 87.531 | 7.940 | 106.630 | 7.654 | 0.0175 |
| 500 μM H_2_O_2_ | 52.252 | 1.322 | 72.2867 | 2.919 | 0.0132 |
| 750 μM H_2_O_2_ | 42.618 | 2.877 | 53.2445 | 2.098 | 0.1687 |

**Minimum data for Fig 5**

HEK293 cells were treated with WT-exosome or Tom40-exosome and the gene expression levels were checked using quantitative real-time PCR. Below we have provided the C_T_ mean, ΔΔC_T_ and 2^-^ ^-ΔΔCt^ (RQ) values. The Relative Quantification (RQ) values were plotted on the Fig 5 graph using GraphPad Prism with SEM.

| Sample | Target | C_T_ mean | ΔC_T_ mean | ΔΔC_T_ | RQ |
| --- | --- | --- | --- | --- | --- |
| WT-exosome treatment | HSPA9 | 20.509323 | 3.908603 | 0.0 | 1.0 |
| Tom40-exosome treatment | HSPA9 | 19.81163 | 3.4847782 | -0.42382494 | 1.3414794 |
| WT-exosome treatment | PDHE1a | 25.250746 | 8.650025 | 0.0 | 1.0 |
| Tom40-exosome treatment | PDHE1a | 24.251406 | 7.9245553 | -0.7254696 | 1.6534387 |
| WT-exosome treatment | aKGDH | 22.512865 | 5.912145 | 0.0 | 1.0 |
| Tom40-exosome treatment | aKGDH | 22.298744 | 5.971892 | 0.05974706 | 0.9594323 |
| WT-exosome treatment | ATP5b | 19.017439 | 2.4167175 | 0.0 | 1.0 |
| Tom40-exosome treatment | ATP5b | 17.735258 | 1.4084072 | -1.0083103 | 2.0115538 |
| WT-exosome treatment | Nrf1 | 22.071844 | 5.9074097 | 0 | 1 |
| Tom40-exosome treatment | Nrf1 | 21.455698 | 5.6546764 | -0.25273323 | 1.1914623 |
| WT-exosome treatment | TFAM | 21.739197 | 5.574763 | 0 | 1 |
| Tom40-exosome treatment | TFAM | 20.925852 | 5.12483 | -0.44993338 | 1.3659772 |
| WT-exosome treatment | Timm44 | 21.928213 | 5.7637787 | 0 | 1 |
| Tom40-exosome treatment | Timm44 | 21.164034 | 5.363012 | -0.400767 | 1.3202096 |
| WT-exosome treatment | CTP1a | 22.97431 | 6.809875 | 0 | 1 |
| Tom40-exosome treatment | CTP1a | 22.497797 | 6.6967773 | -0.11309751 | 1.0815479 |
| WT-exosome treatment | ACADM | 21.308855 | 5.144423 | 0 | 1 |
| Tom40-exosome treatment | ACADM | 20.555172 | 4.75415 | -0.39027342 | 1.3106418 |
| WT-exosome treatment | SOD1 | 21.52774 | 5.3633065 | 0 | 1 |
